# Efficacy and safety of combination therapy Ezetimibe 10/rosuvastatin 40 in Egyptian patients at very high risk of atherosclerotic cardiovascular disease

**DOI:** 10.1186/s43044-025-00655-x

**Published:** 2025-06-11

**Authors:** Mohamed Sobhy, Hala Mahfouz Badran, Mahmoud Hassanein, Samir Rafla, Tarek Zawawy, Amr Zaki, Mohamed Loutfi, Mohamed Sadaka, Sherif Ayad, Amr Kamal, Ahmed Mokhtar, ZEOS investigator group

**Affiliations:** 1 Alexandria University, Alexandria, Egypt; 2 Menoufia University, Shibīn al Kawm, Egypt

**Keywords:** LDL-C reduction, ESC LDL-C targets, Hyperlipidemia, Rosuvastatin-Ezetimibe

## Abstract

**Background:**

Lipid-lowering therapies (LLT) are well-established in reducing cardiovascular complications and improving outcomes in patients with atherosclerotic cardiovascular disease (ASCVD). However, the efficacy of LLT varies across different ethnic and geographical populations, necessitating further investigation.

**Objectives:**

To evaluate the real-world efficacy and safety of the combination therapy of Ezetimibe 10 mg/Rosuvastatin 40 mg in Egyptian patients at very high risk of ASCVD.

**Methods:**

This multicenter, prospective, single-arm, open-label study enrolled adult patients with documented ASCVD or at very high-risk profiles. Lipid parameters, including total cholesterol (TC), LDL-C, HDL-C, non-HDL-C, and triglycerides, were measured at baseline, 6 weeks, and 12 weeks. The primary endpoint was the proportion of patients achieving the ESC LDL-C target (< 55 mg/dL and ≥ 50% reduction). Safety assessments included adverse events, skeletal myopathy, hepatic enzyme alterations, and treatment discontinuation.

**Results:**

A total of 1,854 participants from 25 centers across Egypt completed the 12-week follow-up. The mean age was 56 ± 11 years, with 68% male participants. Hypertension was prevalent in 72.7%, diabetes in 47.6%, smoking in 38.7%, prior ACS in 38.9%, previous percutaneous coronary intervention (PCI) in 43.7%, and prior CABG in 10.4% of the cohort. By week 12, TC decreased by 42.9%, LDL-C by 58.7%, and non-HDL-C by 54%, while HDL-C increased by 9.4%. LDL-C levels < 55 mg/dL were achieved in 35.5% of participants, ≥ 50% LDL-C reduction in 70.3%, and 32.2% met the combined ESC LDL-C goal. Skeletal muscle symptoms occurred in 11.4%, elevated liver enzymes at 0.49%, mostly mild in severity. Treatment discontinuation was minimal (1.9%), with most patients maintaining therapy.

**Conclusion:**

In Egyptian patients at very high risk of ASCVD, the combination of Ezetimibe 10 mg/Rosuvastatin 40 mg over 12 weeks demonstrated substantial LDL-C reduction and high attainment of ESC targets, with an acceptable safety profile.

## Background

Cardiovascular diseases, particularly atherosclerotic cardiovascular disease (ASCVD) and coronary syndromes, remain among the primary causes of death worldwide. One of the key contributors to cardiovascular complications is elevated low-density lipoprotein cholesterol (LDL-C). Various lipid-lowering treatments (LLTs), such as statins, Ezetimibe, bempedoic acid, and proprotein convertase subtilisin/kexin type 9 inhibitors (PCSK9i), have been shown to significantly lower the risk of cardiovascular events and mortality in both primary and secondary prevention settings.[1, 2]

Despite this, much of the available data on LLTs is derived from research focused on predominantly white populations in North America and Europe [3–6]. Studies have demonstrated that responses to LLTs can vary based on ethnic and geographic factors. [7] For instance, findings from the DISCOVERY (DIrect Statin COmparison of LDL-C Values: An Evaluation of Rosuvastatin therapY) study indicated that Chinese patients in Hong Kong exhibited a more pronounced reduction in LDL-C levels with Rosuvastatin compared to their counterparts in other Asian and Western regions (52.8% vs. 41.2%).[8]

These disparities in LLT effectiveness are frequently linked to genetic variations [7]. Genetic polymorphisms can influence individual responses to statins [9], and a particular variant in Niemann-Pick C1-like 1 (NPC1L1), [10] which is responsible for cholesterol transport and serves as the target for Ezetimibe, may enhance the drug’s LDL-C-lowering effects. Additionally, socioeconomic elements such as income, education, and regional economic conditions, health care system, patients'awareness, patients'adherence to treatment can also impact the success of LLT treatments. [11, 12]

Egypt has one of the highest cardiovascular diseases (CVD) mortality rates globally, with a particularly high burden of coronary artery disease (CAD). [13] According to the World Health Organization (WHO), Egypt ranks second in age-standardized CAD incidence rates, with 759.9 cases per 100,000 people. [13, 14] It also has one of the highest age-standardized death rates from CAD, reaching 359 per 100,000, and the highest disability-adjusted life years (DALY) rate due to CAD (6,986 per 100,000). In 2014, CAD was responsible for 23.14% of all deaths in Egypt, [15, 16] and the age-adjusted death rate was 186.36 per 100,000, ranking Egypt 23rd globally.

Dyslipidemia prevalence in Egypt is 19.2%. The Global Burden of Disease (GBD) 2019 study attributed 46.8% of CAD deaths to high LDL-C, with the highest burden observed in Egypt, Syria, and Oman. [14, 17]

Observational trials indicate that accomplishing LDL-C targets is suboptimal in countries outside of Western Europe. [18, 19] In the Middle East and North Africa region, only 33–50% of patients treated reach LDL-C targets, with extensive difference depending on CVD risk groups. [20–23] In Iran, for instance, only 43.4% of patients achieved their LDL-C targets, with achievement rates varying significantly across risk categories. [22]

While geographic and ethnic differences in risk profiles are acknowledged, there is limited data on the response of Egyptian patients to LLTs, particularly regarding LDL-C target achievement in those with documented ASCVD.

This research examines the real-world efficacy of the combination therapy of Ezetimibe/Rosuvastatin (10/40 mg) (Ezet/Rousuv 10/40) over three months in reducing LDL-C and achieving the European Society of Cardiology (ESC) LDL-C goals in Egyptian patients at very high risk of ASCVD. Additionally, this investigation seeks to evaluate the safety and tolerability of the combination therapy in this specific Egyptian population.

## Patients and methods

This is a multicenter, single-arm, open-label prospective study conducted from November 2023 to December 2024, and patients included were followed up for a period of 12 weeks. The study is accomplished in accordance with the ethical principles in the Declaration of Helsinki and approved by the ethical committee of Alexandria University. Written informed consent was received from all patients before enrollment.

### Study procedures

Patients having the inclusion criteria were recruited from 25 centers from different governmental area in Egypt including, private clinics, university and governmental hospitals. During the whole study period, patients treated in the routine clinical setting; all treatment decisions followed the general clinical practice and were not influenced by the study protocol.

### Inclusion criteria

Patients > 18 years with any of the following:"Established ASCVD cases include a history of myocardial infarction (MI), unstable or stable angina, coronary interventions (PCI, CABG), peripheral arterial revascularization, stroke, or TIA. Definitive ASCVD evidence from imaging encompasses significant plaque detection via coronary angiography or CT scan (≥ 50% stenosis in at least two major epicardial arteries) or carotid ultrasound findings. [23–25] Additionally, Type-2 diabetes mellitus (T2DM) with target organ damage, the presence of three or more major risk factors, and/or a calculated SCORE of ≥ 10% for a 10-year fatal CVD risk were considered."

### Exclusion criteria

Patients with a documented history of statin-induced severe adverse effects or hypersensitivity reactions to statins, recent acute coronary syndrome (ACS), or planned coronary revascularization were excluded. Additional exclusion criteria included congestive heart failure, active malignancy, hypothyroidism, active hepatic disease (defined as alanine aminotransferase (ALT) and/or aspartate aminotransferase (AST) levels exceeding three times the upper limit of normal), renal impairment characterized by severe chronic kidney disease (CKD) (estimated glomerular filtration rate [eGFR] < 30 mL/min/1.73 m^2^), patients undergoing renal dialysis, pregnancy, or prior use of statin/Ezetimibe combination therapy before the initial assessment. Furthermore, patients presenting with fasting LDL-C concentrations ≤ 55 mg/dL at the initial visit (Visit 1) were also excluded.

**Study intervention**: Eligible patients received a fixed-dose combination therapy consisting of Ezetimibe 10 mg/Rosuvastatin 40 mg, administered as a single tablet once daily for a duration of 12 weeks.

#### Baseline assessments and clinical evaluations

All patients underwent comprehensive medical history assessment and physical examination, with documentation of cardiovascular risk factors, smoking status, hypertension (defined as blood pressure ≥ 140/90 mm Hg or ongoing antihypertensive medication), T2DM, and a prior occurrence of ACS, percutaneous coronary intervention (PCI), or coronary artery bypass grafting (CABG).

#### Laboratory assessments

Fasting blood samples were collected at baseline (study entry), week 6, and week 12. The following biochemical parameters were measured:

- Lipid profile: LDL-C (mg/dL), high-density lipoprotein cholesterol (HDL-C) (mg/dL), non-HDL cholesterol (mg/dL), total cholesterol (TC) (mg/dL), triglycerides (TG) (mg/dL). Serum creatinine, hepatic function tests: AST (U/L), ALT (U/L), creatine phosphokinase (CPK)
- All laboratory evaluations, including hematology and comprehensive blood chemistry analysis, were conducted in a fasting state at baseline, week 6, and week 12 at a central laboratory to ensure consistency and accuracy of biochemical assessments.

#### Safety and compliance assessments

Adverse events (AEs), including myalgia, gastrointestinal tract symptoms like nausea, vomiting, elevated liver enzyme levels, and angina pectoris, were recorded. Discontinuation of therapy due to adverse effects was documented, with specific reasons noted. The safety assessment covered the occurrence of treatment-related adverse events, drug reactions, and serious complications throughout the study. Adverse events were categorized as mild, moderate, or severe according to patient-reported experiences.

Compliance with the prescribed therapy was evaluated in week 6 and week 12 by calculating the number of returned tablets. Patients were deemed compliant if they took a minimum of 80% of the prescribed doses.

**Study endpoints:** The primary efficacy endpoint was the proportion of patients achieving LDL-C levels < 55 mg/dL and/or ≥50% reduction after 12 weeks of treatment. The key secondary endpoint analysis included the proportion of participants who achieved an LDL-C reduction of more than 50% from baseline without discontinuation due to adverse events. Additional secondary endpoints included changes in HDL-C, TC, TG, and non-HDL cholesterol levels over the study period.

### Statistical analysis

Data management and statistical analysis were conducted using SPSS version 28 (IBM, Armonk, New York, United States). Normality of quantitative data was assessed using the Shapiro–Wilk test and direct data visualization methods. Depending on normality distribution, quantitative data were summarized as means with standard deviations or as medians with ranges. Categorical data were presented as frequencies and percentages. Comparisons of quantitative data across different follow-up time points were performed using repeated measures ANOVA, with post-hoc analyses adjusted for multiple comparisons. All statistical tests were two-sided, and a p-value of < 0.05 was considered statistically significant.

## Results

### Demographic and clinical characteristics

**Fig. 1:**
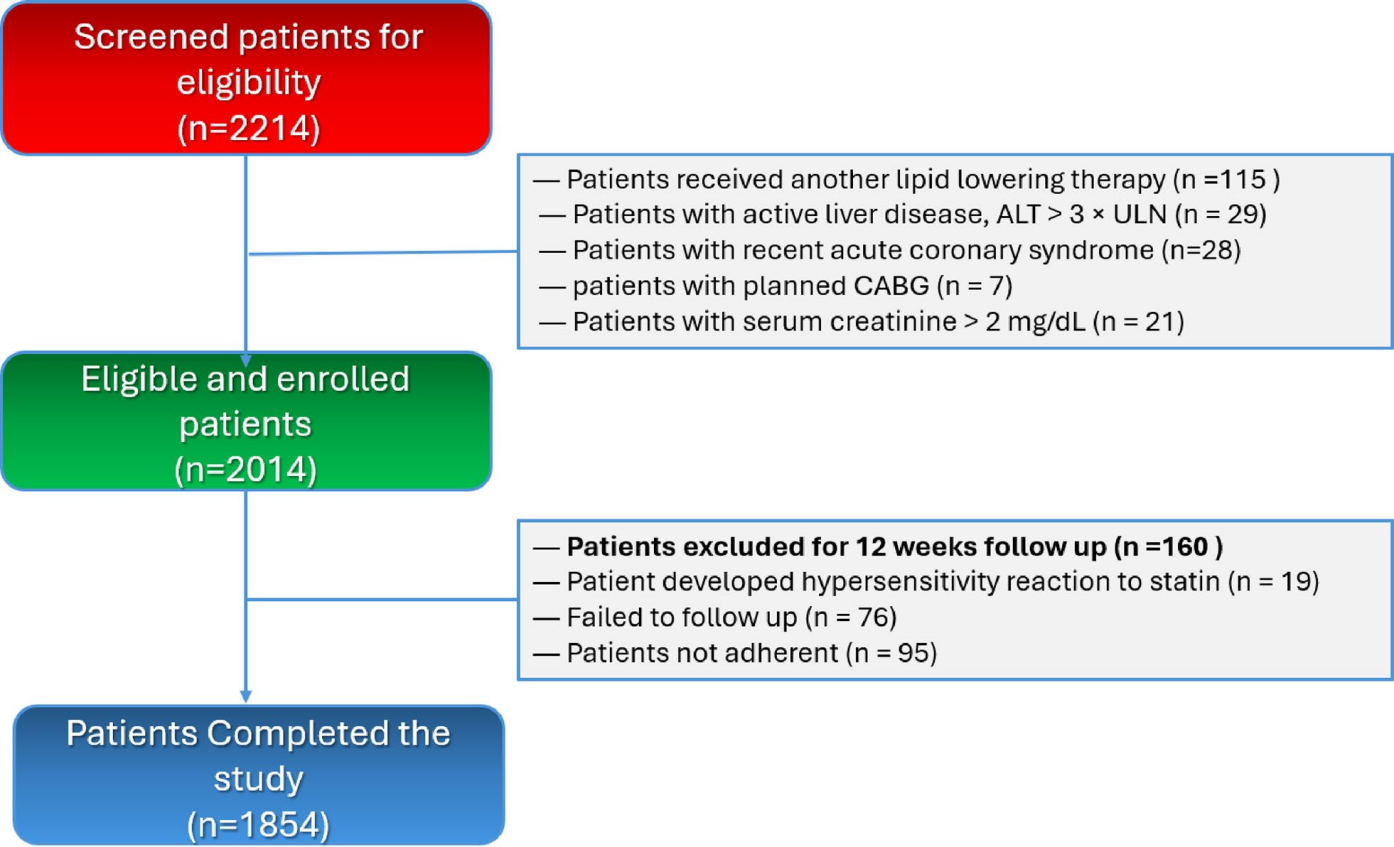
A flowchart of patient inclusion, exclusions, and study completion. ALT: alanine transaminase; ULN: upper limit of normal

A total of 2,214 patients were screened for eligibility in the study. Of these, 200 individuals were excluded due to various criteria: 115 patients were undergoing treatment with an alternative statin-based combination therapy in the last 3 months, 29 had active hepatic pathology, 28 had a recent history of ACS within the past month, 7 were scheduled for CABG, and 21 exhibited serum creatinine levels exceeding 2 mg/dL (Fig. 1).

Following enrollment and throughout the six-week follow-up period, an additional 160 patients were excluded. Specifically, 19 individuals experienced intolerance/hypersensitivity reactions, 76 were lost to follow-up, and 95 demonstrated non-adherence to the prescribed combination therapy.

The studied patients had a mean age of 56 ± 11 years, 68% were male and a mean BMI of 30.2 ± 5.5. Risk factors were prevalent, with 47.6% (*n* = 882) of the participants having diabetes mellitus (DM), 72.7% (*n* = 1347) having hypertension (HTN), 50% were obese (*n* = 925) and 38.7% (*n* = 718) being smokers. Documented ASCVD were also prominent, with 38.9% (*n* = 722) having prior acute coronary syndrome (ACS), 43.7% (*n* = 811) having undergone percutaneous coronary intervention (PCI), and 10.4% (*n* = 193) having a history of CABG. The median duration of dyslipidemia was 3 years, and 69.5% (*n* = 1288) had previously been treated. The median duration of treatment was 2 years. Table 1

**Table 1:** Demographic and clinical characteristics of the study population

General characteristics		
Age (years)	Mean ± SD	56 ± 11
Male Female	*n* (%) *n* (%)	1261(68%) 593(32%
BMI (Kg/m2) Normal weight (< 25) Overweight (> 25 < 30) Obese (> 30 < 40) Morbid Obese (> 40)	Mean ± SD *n* (%) *n* (%) *n* (%) *n* (%)	30.2 ± 5.5 180 (9.7) 749 (40.4%) 611(33%) 314(16.9)
T2DM	*n* (%)	882 (47.6)
HTN	*n* (%)	1347 (72.7)
Smoking	*n* (%)	718 (38.7)
CAD	*n* (%)	722 (38.9)
PCI	*n* (%)	811 (43.7)
CABG	*n* (%)	193 (10.4)
Dyslipidemia duration (years)	Median	3 (0–40) *
Previous drug for dyslipidemia	*n* (%)	1288 (69.5)
Duration of treatment (years)	Median	2 (0.1–25)

SD: Standard deviation; BMI: Body mass index; T2DM: type-2 Diabetes mellitus; n: Number; HTN: Hypertension; CAD: Coronary artery disease; PCI: Percutaneous coronary intervention; CABG: Coronary artery bypass grafting; *: 0 indicates naive patients

### Lipid profile and liver enzymes

**Fig. 2:**
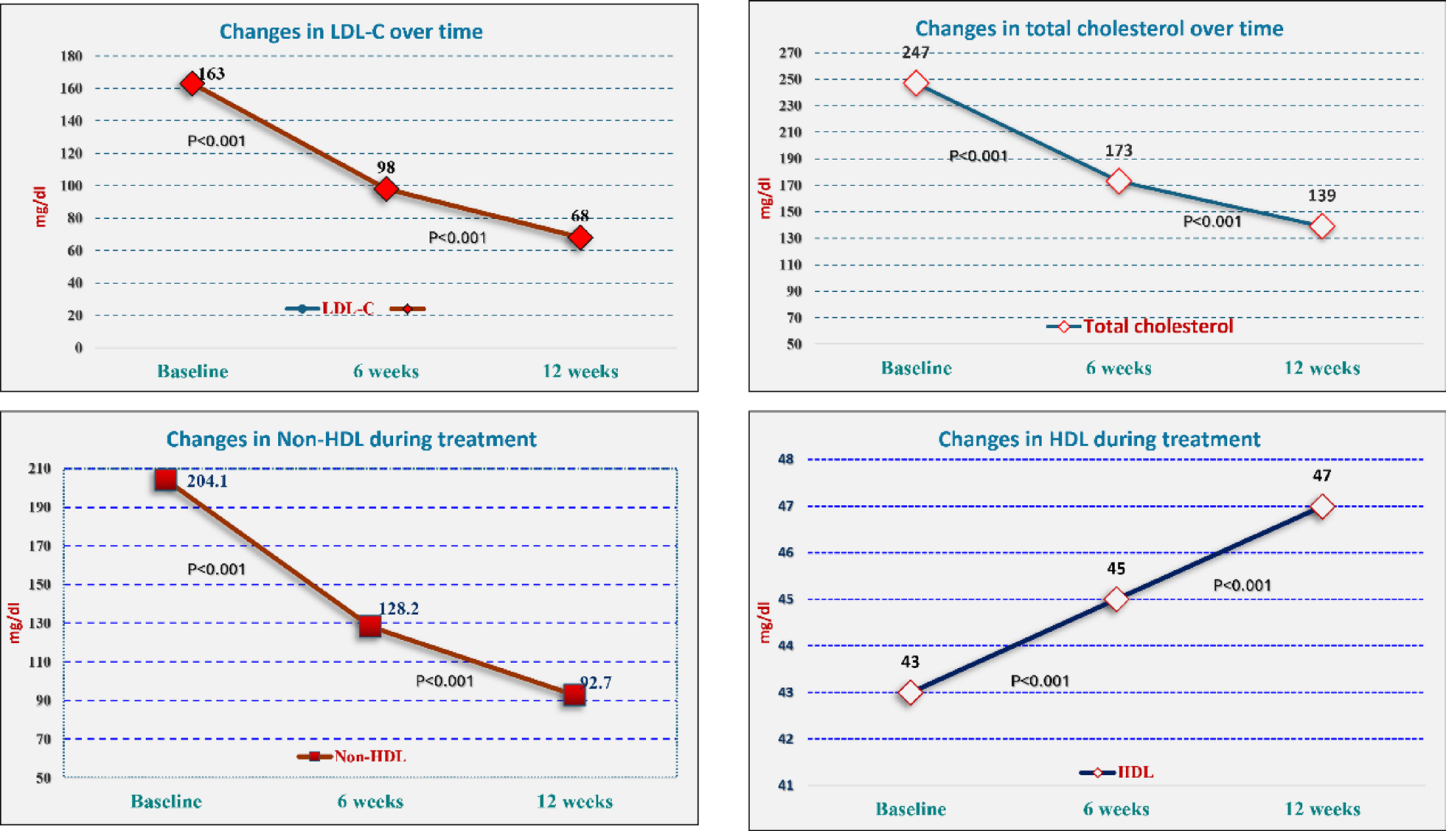
The changes in lipid profile over time. There are significant changes from baseline to 6 weeks and to 12 weeks. The trend demonstrates the effectiveness of Ezetimibe/Rosuvastatin combination therapy in lowering total lipid levels over the study period

Changes were observed in lipid profile and liver enzyme levels over 12 weeks, with all values demonstrating statistically significant differences from baseline (*P* < 0.001). Table 2 Total cholesterol levels decreased significantly from a baseline mean of 247 ± 56 mg/dl to 173 ± 41 mg/dl in 6 weeks and 139 ± 32 mg/dl at 12 weeks, showing a median percent change of −28.2% and −42.9%, respectively (Fig. 2).

**Table 2:** Baseline and follow-up assessment of lipid profile and hepatic enzyme levels

Parameter			*P*-value
*Total cholesterol (mg/dl)*			
Baseline	Mean ± SD	247 ± 56	< 0.001*
At 6 weeks	Mean ± SD	173 ± 41^**T**^	
% change at 6 weeks	Median (range)	− 28.2 (− 72.2–74.2)	
At 12 weeks	Mean ± SD	139 ± 32^**T**^	
% change at 12 weeks	Median (range)	− 42.9 (− 80.1–11.9)	
*LDL-C (mg/dl)*			
Baseline	Mean ± SD	163 ± 51	< 0.001*
At 6 weeks	Mean ± SD)	98 ± 36^**T**^	
% change at 6 weeks	Median (range)	− 38.3 (− 93.9–156.5)	
At 12 weeks	Mean ± SD	68 ± 27^**T**^	
% change at 12 weeks	Median (range)	− 58.7 (− 96.2–40.2)	
*HDL-C (mg/dl)*			
Baseline	Mean ± SD	43 ± 15	< 0.001*
At 6 weeks	Mean ± SD	45 ± 11^**T**^	
% change at 6 weeks	Median (range)	5.3 (− 82.9–262.5)	
At 12 weeks	Mean ± SD	47 ± 12^**T**^	
% change at 12 weeks	Median (range)	9.4 (−85—335)	
*Non-HDL-C (mg/dl)*			
Baseline	Mean ± SD	204.1 ± 56.8	< 0.001*
At 6 weeks	Mean ± SD	128.2 ± 41.3^**T**^	
% change at 6 weeks	Median (range)	− 35.6 (− 94.4–100)	
At 12 weeks	Mean ± SD	92.7 ± 33.5^**T**^	
% change at 12 weeks	Median (range)	− 54 (− 30.4–109.4)	
*TG (mg/dl)*			
Baseline	Mean ± SD	193 ± 88	< 0.001*
At 6 weeks	Mean ± SD	151 ± 53^**T**^	
% change at 6 weeks	Median (range)	− 15.4 (− 82.5–224.3)	
At 12 weeks	Mean ± SD	132 ± 42^**T**^	
% change at 12 weeks	Median (range)	− 26.1 (− 86.2–402.7)	
*AST (U/L)*			
Baseline	Mean ± SD	28 ± 11	< 0.001*
At 6 weeks	Mean ± SD	30 ± 12^**T**^	
% change at 6 weeks	Median (range)	8.7 (− 70.5–950)	
At 12 weeks	Mean ± SD	32 ± 12^**T**^	
% change at 12 weeks	Median (range)	14.3 (− 66.7–950)	
*ALT (U/L)*			
Baseline	Mean ± SD	27 ± 11	< 0.001*
At 6 weeks	Mean ± SD	30 ± 11^**T**^	
% change at 6 weeks	Median (range)	7.9 (− 88.9–330.4)	
At 12 weeks	Mean ± SD	32 ± 12^**T**^	
% change at 12 weeks	Median (range)	13.9 (− 91.4–440)	

^*^Significant *P*-value; ^**T**^Significant from baseline; LDL.C: Low-density lipoprotein cholesterol; HDL.C: High-density lipoprotein cholesterol; Non-HDL-C: Non-high-density lipoprotein cholesterol; TG: Triglycerides; AST: Aspartate aminotransferase; ALT: Alanine aminotransferase *: Bolded values indicate statistically significant differences from baseline measurements, with P-values <0.001, using two- sided t- test

LDL-C levels decreased from a mean of 163 ± 51 mg/dl at baseline to 98 ± 36 mg/dl in 6 weeks, and further to 68 ± 27 mg/dl at 12 weeks, with median percentage changes of − 38.3% and − 58.7%, respectively.

HDL-C increased slightly from a mean of 43 ± 15 mg/dl at baseline to 45 ± 11 mg/dl in 6 weeks, and 47 ± 12 mg/dl at 12 weeks, with median percent changes of 5.3% and 9.4%, respectively.

Non-HDL-C levels decreased from a mean of 204.08 ± 56.82 mg/dl at baseline to 128.19 ± 41.3 mg/dl in 6 weeks, and 92.71 ± 33.48 mg/dl at 12 weeks, with median percent changes of −35.63% and −54.01%, respectively.

**Fig. 3:**
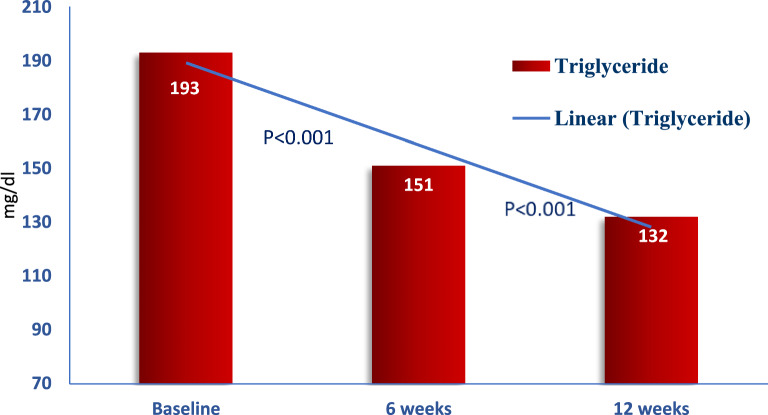
Changes in triglyceride during treatment

Triglycerides (TG) decreased from a mean of 193 ± 88 mg/dl at baseline to 151 ± 53 mg/dl at 6 weeks, and 132 ± 42 mg/dl at 12 weeks, with median percent changes of −15.4% and −26.1%, respectively. (Fig. 3).

Liver enzymes (AST and ALT) also showed slight increases, with AST levels rising from a mean of 28 ± 11 U/L at baseline to 30 ± 12 U/L at 6 weeks and 32 ± 12 U/L at 12 weeks. ALT levels increased from a mean of 27 ± 11 U/L at baseline to 30 ± 11 U/L in 6 weeks and 32 ± 12 U/L at 12 weeks, reflecting median percent changes of 8.7% and 14.3% for AST, and 7.9% and 13.9% for ALT.

### LDL-C endpoint at 12 weeks

**Fig. 4:**
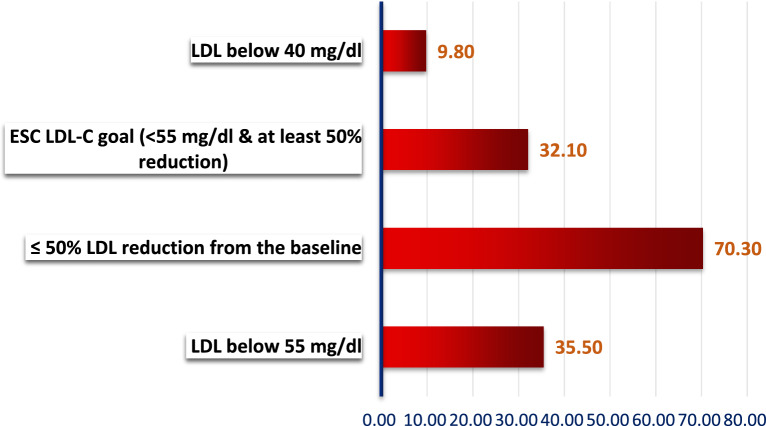
Percentage of patients achieved LDL-C goal at the end of the study

Among the studied patients, 35.5% (*n* = 627) achieved an LDL level below 55 mg/dl. A significant proportion, 70.3% (*n* = 1240), achieved at least a 50% reduction in LDL from baseline. Additionally, 32.2% (*n* = 568) accomplished the ESC LDL-C goal of achieving LDL below 55 mg/dl and a 50% reduction from baseline. A smaller subset, 9.7% (*n* = 171), achieved an LDL level below 40 mg/dl. Table 3***, ***Fig. 4

**Table 3:** LDL-c levels at the 12-week endpoint in the study population

Goal	*n* (%)
LDL below 55 mg/dl	627 (35.5)
≥ 50% LDL reduction from the baseline	1240 (70.3)
ESC LDL-C goal (< 55 mg/dl & at least 50% reduction)	568 (32.2)
LDL below 40 mg/dl	171 (9.7)

n: Number; LDL: low-density lipoprotein; ESC: European society of cardiology

### Side effects and treatment discontinuation

Skeletal muscle symptoms were reported by 11.4% (*n* = 212) of patients, while 0.49% (*n* = 9) experienced mild to moderate elevation of liver enzymes, only 1 patient (0.05%) had elevation of > 3 times the upper limit of normal without symptoms. Other side effects [including hematuria, proteinuria, and increased serum creatinine > 50% of baseline value] were reported by 1.6% (*n* = 30) of patients. Regarding treatment discontinuation, 1.9% (*n* = 35) of patients discontinued the treatment entirely because of side effects, while 0.1% (*n* = 2) temporarily discontinued treatment (Table 4). The vast majority, 98% (*n* = 1817), continue the treatment without interruption. No severe or serious side effects were reported that require hospitalization or lead to death.

**Table 4:** Adverse effects and treatment discontinuation in the study population

	*n* (%)
Skeletal muscle symptoms	212 (11.4)
Liver enzymes	9 (0.49)
Other side effects^#^	1 (0.05)
Treatment discontinuation* n* (%)	35 (1.9)
Temporary discontinuation* n* (%)	2 (0.1)
No discontinuation* n* (%)	1817 (98)

^#^: Other side effects [including hematuria, proteinuria, and increased serum creatinine > 50% of baseline value]

## Discussion

This study represents the first large-scale investigation into the efficacy and safety of Ezetimibe 10 mg/Rosuvastatin 40 mg combination therapy in Egyptian patients at very high risk of ACVD. It is one of the most efficacious combinations currently available for lipid management in this patient population.

The findings of this study demonstrate that the combination therapy of Ezetimibe 10 mg/Rosuvastatin 40 mg remarkably decreased LDL-C, TC, non-HDL-C, and TG levels, while increasing HDL-C levels after 12 weeks of therapy. Notably, 35.5% of patients attained a reduction in LDL-C to below 55 mg/dL, 70% exhibited a greater than 50% reduction in LDL-C from baseline, and 32% achieved the ESC LDL-C target of < 55 mg/dL, in addition to a > 50% reduction from baseline. The therapy was well tolerated, with only 13.6% of patients experiencing mild adverse effects, including myalgia, elevated liver enzymes, and increased creatinine, which did not necessitate treatment discontinuation, except in 1.9% of patients.

Rosuvastatin, a strongly compelling inhibitor of HMG-CoA reductase, has been demonstrated to reduce LDL-C concentrations by up to 55%, [26, 27] elevate HDL-C by nearly 6%, and lower triglyceride (TG) levels by around 15%. Additionally, it contributes to the regression of atherosclerotic plaque lipid content [28] and exerts multiple pleiotropic effects, including anti-inflammatory properties, endothelial function enhancement, and antioxidant activity [29, 30]. Due to its hydrophilic nature, Rosuvastatin is associated with a lower risk of myopathy and rhabdomyolysis. Its metabolism is minimally dependent on the Cytochrome P450 enzyme system, with only 10% of the administered dose undergoing hepatic metabolism, while the majority (90%) is excreted via the biliary route, thereby minimizing the potential for pharmacokinetic drug interactions.

Ezetimibe, a unique cholesterol absorption inhibitor, exerts anti-inflammatory effects through the selective inhibition of NPC1L1 protein, leading to a 67% reduction in intestinal cholesterol uptake. This mechanism contributes to a 15–20% decrease in LDL-C levels and a modest 3% increase in HDL-C concentrations, with no significant impact on TG levels. [31, 32]

While statins reduce endogenous cholesterol synthesis in the liver, this can trigger increased cholesterol absorption as a compensatory mechanism, potentially reducing the efficiency of statins. The adding of Ezetimibe mitigates this compensatory increase, thereby enhancing statin efficacy. Furthermore, the combination of Ezetimibe and statins results in a more significant reduction in high-sensitivity C-reactive protein (hs-CRP) by approximately 10% compared to statin monotherapy. [32]

Several investigations have evaluated the combination of Ezet/Rousu to Rosuvastatin monotherapy in high-risk patients or those with preexisting CVD. The EXPLORER trial, conducted in multiple countries including United States, Germany, Austria, Switzerland, and South Africa [26] randomized 469 high-risk patients to either Rosuvastatin alone or the combination therapy for 6 weeks. The combination therapy significantly outperformed Rosuvastatin monotherapy, with a 69.8% drop in LDL-C, compared to 57.1% with Rosuvastatin alone (*p* < 0.001). Additionally, a higher proportion of patients in the combination arm accomplished the most favorable LDL-C target of < 70 mg/dL (79.6% versus 35%).

In our study, the Ezet/Rousu 10/40 combination therapy tempted a 38% reduction in LDL-C in 6 weeks, and a 58.7% reduction in 12 weeks, with mean LDL-C levels decreasing from 163 mg/dL to 98 mg/dL at 6 weeks and from 163 mg/dL to 68 mg/dL at 12 weeks. While the reduction in LDL-C in this study was somewhat lower than that observed in the EXPLORER trial (69.8%), it is noteworthy that our patient population had considerably higher baseline LDL-C levels, and our study aimed to attain an LDL-C goal of < 55 mg/dL, which is more stringent than the target of < 70 mg/dL in the EXPLORER trial.

The study also demonstrated a lesser reduction in non-HDL-C (54% versus 65%), TC (42% versus 51%), and TG (26% versus 35%) levels, as well as a smaller increase in HDL-C (9% versus 11%), compared to the EXPLORER trial. [26]

In a similar vein, the study by Yang et al. (2020) directed a randomized, double-blind, placebo-controlled trial in Koreans (*n* = 337) with moderate to high CV risk, in which patients receiving Rosuvastatin/Ezetimibe combination therapy showed significantly better lipid-lowering effects than those on monotherapy, with a 59.5% reduction in LDL-C and 90.7% of participants achieving their LDL-C target. [33] In comparison, our study observed a 58.7% reduction in LDL-C, and 70.3% of participants reached their LDL-C target. Musculoskeletal adverse events were reported more frequently in our study (11.7%) compared to Yang et al.'s study (2.4%).

Similarly, the MRS-ROZE trial [34] conducted in South Korea (2020) demonstrated a significant reduction in LDL-C by 59.1% in the combination therapy group in comparison with 49.4% with Rosuvastatin alone, with 94.1% of participants achieving the LDL-C goal, a higher proportion than in our study. However, both studies showed comparable safety profiles with no serious adverse events.

The I-ROSETTE trial [35], conducted in Japan, also highlighted the efficacy of Rosuvastatin/Ezetimibe combination therapy, with an LDL-C reduction of 82.0% in the combination group versus 64.4% in the monotherapy group. Musculoskeletal symptoms were less frequent in this study (2%) than in our study (11.4%).

A multicenter, randomized, double-blind clinical trial conducted in South Korea [35] evaluated the effectiveness and safety of Rosuvastatin/Ezetimibe combination treatment in comparison with Rosuvastatin monotherapy over an 8-week period. The study enrolled 712 patients with hypercholesterolemia and baseline LDL-C levels below 250 mg/dL. Participants were randomized to receive either Rosuvastatin/Ezetimibe at doses of 5/10 mg, 10/10 mg, or 20/10 mg, or Rosuvastatin alone at equivalent doses of 5, 10, and 20 mg [35]. The combination therapy demonstrated a significantly greater reduction in LDL-C levels compared to monotherapy (56.4% vs. 45.18%, *p* < 0.01). Furthermore, a higher percentage of patients in the combination group achieved their LDL-C target (94.15% vs. 86.63%, *p* < 0.01). Adverse event rates were similar between groups, though elevations in alanine aminotransferase (ALT) levels were observed more frequently in the combination therapy cohort (1.57%) than in the monotherapy group (1.05%). [36]

A randomized clinical trial conducted in Japan by Torimoto et al. [37] investigated the lipid-lowering efficacy of Rosuvastatin/Ezetimibe combination therapy compared to Rosuvastatin monotherapy in individuals with type II diabetes mellitus and baseline LDL-C levels exceeding 80 mg/dL. A total of 79 patients were randomly assigned to receive either Rosuvastatin 2.5 mg/Ezetimibe or Rosuvastatin 5 mg alone.

During the 12-week follow-up, LDL-C reduction was significantly greater in the combination therapy group (31%) compared to monotherapy (15.6%, *p* < 0.001). Additionally, a higher proportion of patients receiving the combined regimen achieved their LDL-C target (89.7% vs. 58.3%). No cases of elevated creatine kinase or abnormal liver function tests were reported. However, these reductions were lower than those observed in our study, where LDL-C levels decreased by 58% over 12 weeks, though 1.6% of patients developed proteinuria and exhibited increased serum creatinine levels.

The safety and efficacy profile of Rosuvastatin/Ezetimibe combination therapy has been well-established across multiple populations and is comparable to other combination therapies, including the Ezetimibe/Simvastatin combination [38]. This regimen offers superior LDL-C lowering, increased HDL-C, and pleiotropic benefits over monotherapy. It is widely recommended as an add-on therapy for patients who cannot achieve their LDL-C goals with statins alone, as evidenced by several international guidelines. [39–41]

In resource-constrained settings, such as Egypt, the cost-effectiveness of Rosuvastatin/Ezetimibe combination therapy presents a viable alternative to high-cost lipid-lowering agents, including PCSK9 inhibitors, which necessitate injectable administration. [38, 42, 43] While PCSK9 inhibitors exhibit superior efficacy in reducing LDL-C and improving cardiovascular outcomes, their high cost renders them impractical for widespread use in low- and middle-income countries. [44, 45]

Our study holds significant clinical relevance, offering valuable data on the efficacy and safety of combination therapy in a high-risk Egyptian cohort. However, variations in geographic and ethnic factors, including baseline characteristics and socioeconomic disparities, may influence both treatment adherence and lipid-lowering efficacy. Prior research has indicated that individuals from nonwhite racial backgrounds and lower-income populations demonstrate lower adherence to statin therapy, potentially contributing to observed differences in LDL-C reduction and therapeutic response across diverse ethnic groups. [45]

## Study limitations

This study has some limitations, primarily being a single-arm trial, which was designed to evaluate the real-world efficacy and safety of Ezetimibe 10 mg/Rosuvastatin 40 mg combination therapy in Egyptian patients. Single-arm trials offer lower costs and shorter timelines; however, they are less robust than randomized controlled trials (RCTs) in determining causal relationships. Additionally, comparison to other populations or ethnic groups with varying treatment responses would be beneficial in elucidating the impact of genetic and environmental factors on therapy outcomes.

## Conclusion

In Egyptian patients at an elevated risk for atherosclerotic cardiovascular disease (ASCVD), the administration of a combined regimen consisting of Rosuvastatin 40 mg and Ezetimibe 10 mg has demonstrated significant efficacy in reducing low-density lipoprotein cholesterol (LDL-C). This therapeutic approach serves as a viable alternative for individuals who fail to achieve target LDL-C levels with statin monotherapy. Although the observed reduction in LDL-C appears to be marginally lower than that reported in studies involving other ethnic populations, the combination therapy exhibits favorable tolerability, a low incidence of adverse effects, and a convenient once-daily dosing schedule. Additional investigations focusing on clinical endpoints, such as major adverse cardiovascular events (MACE), are warranted to elucidate the long-term benefits and optimize the clinical application of this treatment.

## Data Availability

No datasets were generated or analysed during the current study.

## Abbreviations

ASCVD: Atherosclerotic cardiovascular disease
LDL-C: Low-density lipoprotein cholesterol
LLTs: Lipid-lowering therapies
CAD: Coronary artery disease
HDL-C: High-density lipoprotein cholesterol
TC: Total cholesterol
TG: Triglyceride
ACS: Acute coronary syndrome
T2DM: Type-2 diabetes mellitus
