# Correction: Efficacy and safety of combination therapy Ezetimibe 10/rosuvastatin 40 in Egyptian patients at very high risk of atherosclerotic cardiovascular disease

**DOI:** 10.1186/s43044-025-00690-8

**Published:** 2025-10-09

**Authors:** Mohamed Sobhy, Hala Mahfouz Badran, Mahmoud Hassanein, Samir Rafla, Tarek Zawawy, Amr Zaki, Mohamed Loutfi, Mohamed Sadaka, Sherif Ayad, Amr Kamal, Ahmed Mokhtar, ZEOS investigator group

**Affiliations:** 1 Alexandria University, Alexandria, Egypt; 2 Menoufia University, Shibīn Al Kawm, Egypt

**Correction**: **The Egyptian Heart Journal (2025) 77:59** 10.1186/s43044-025-00655-x

Following publication of the original article [1], the authors identified errors in their article.

The present study evaluated the efficacy and safety of combination therapy with ezetimibe (10 mg) and rosuvastatin (40 mg) in Egyptian patients at very high risk of atherosclerotic cardiovascular disease. Safety outcomes included the assessment of adverse events, skeletal myopathy, hepatic enzyme alterations, and treatment discontinuation. In the published version, the number of patients with elevated liver enzymes was incorrectly reported as 91 (4.9%). The correct figure is 9 patients (0.49%) with mild to moderate elevation of liver enzymes, while only 1 patient (0.05%) experienced an elevation exceeding three times the upper limit of normal. Accordingly, the percentages will be corrected in six instances (as highlighted in the attached file). In addition, Table 4 and typographical errors identified in Table 3 are corrected as per the table below.

**Table:** 

Page-Line	Before	After
Page 1- line 23	Elevated liver enzymes in 4.9%,	Elevated liver enzymes at 0.49%,
Page 3-line 56	LDL-C levels < 55 mg/dl	LDL-C levels < 55 mg/dl and/or ≥ 50% reduction
Page 5- line 28	While 4.9% (n = 91) experienced clinimodified.vant elevated liver enzymes (> 3 upper limit of normal)	While 0.49% (n = 9) experienced mild to moderate elevation of liver enzymes, only 1 patient (0.05%) had elevation of > 3 times the upper limit of normal without symptoms
Page 6- line 6	With only 17.9% of patients experiencing	With only 13.6% of patients experiencing

Incorrect Table 3

**Table 3:** LDL-C Levels at the 12-Week Endpoint in the Study Population

Goal	N (%)
LDL below 55 mg/dl	627 (35.5)
≤ 50% LDL reduction from the baseline	1240 (70.3)
ESC LDL-C goal (< 55 mg/dl & at least 50% reduction)	568 (32.2)
LDL below 40 mg/dl	171 (9.7)

n: Number; LDL: Low-density lipoprotein; ESC: European Society of Cardiology

Correct Table 3

**Table 3:** LDL-C Levels at the 12-Week Endpoint in the Study Population

Goal	n (%)
LDL below 55 mg/dl	627 (35.5)
≥ 50% LDL reduction from the baseline	1240 (70.3)
ESC LDL-C goal (< 55 mg/dl & at least 50% reduction)	568 (32.2)
LDL below 40 mg/dl	171 (9.7)

n: Number; LDL: Low-density lipoprotein; ESC: European Society of Cardiology

Incorrect Table 4

**Table 4:** Adverse effects and treatment discontinuation in the study population

	n (%)
Skeletal muscle symptoms	212 (11.4)
Liver enzymes	91 (4.9)
Other side effects	30 (1.6)
Treatment discontinuation n (%)	35 (1.9)
Temporary discontinuation n (%)	2 (0.1)
No discontinuation n (%)	1817 (98)

ULN: upper limit of normal; Other side effect: [including hematuria, proteinuria, and increased serum creatinine 50% of baseline value]

Correct Table 4

**Table 4:** Adverse effects and treatment discontinuation in the study population

	n (%)
Skeletal muscle symptoms	212 (11.4)
Liver enzymes elevation 3 times compared to baseline	9 (0.49)
Liver enzymes > 3 ULN (Clinically relevant elevation)	1 (0.05)
Other side effects	30 (1.6)
Treatment discontinuation n (%)	35 (1.9)
Temporary discontinuation n (%)	2 (0.1)
No discontinuation n (%)	1817 (98)

ULN: upper limit of normal; Other side effect: [including hematuria, proteinuria, and increased serum creatinine > 50% of baseline value]

The original article [1] has been corrected.
